# Neurocognitive function in procedures correcting severe aortic valve stenosis: patterns and determinants

**DOI:** 10.3389/fcvm.2024.1372792

**Published:** 2024-04-12

**Authors:** L. Ranucci, L. Brischigiaro, V. Mazzotta, M. Anguissola, L. Menicanti, F. Bedogni, M. Ranucci

**Affiliations:** ^1^ Department of Cardiovascular Anesthesia and Intensive Care, IRCCS Policlinico San Donato, Milan, Italy; ^2^ Department of Cardiac Surgery, IRCCS Policlinico San Donato, Milan, Italy; ^3^ Department of Cardiology, IRCCS Policlinico San Donato, Milan, Italy

**Keywords:** aortic valve stenosis, TAVI, SAVR, neurocognitive function, transfusions

## Abstract

**Background:**

Neurocognitive changes occurring after a surgical aortic valve replacement (SAVR) or transcatheter aortic valve implantation (TAVI) procedure for the correction of severe aortic stenosis (AS) have not been widely addressed and, if addressed, have produced conflicting results. The purpose of this study is to identify the pre-procedural neurocognitive pattern and its determinants in a setting of elderly (>65 years) patients with severe AS undergoing SAVR or TAVI and the changes occurring at a 2–3 month follow-up.

**Methods:**

This was a prospective cohort study included in the Italian Registry on Outcomes in Aortic Stenosis Treatment in Elderly Patients. Patients were assessed both before and after (2–3 months) the procedure using the Montreal Cognitive Assessment (MoCA) test. Data on periprocedural demographics, clinical factors, and outcome measures were collected.

**Results:**

Before the procedure, 70% of the patients demonstrated a MoCA score <23 points, which was indicative of cognitive dysfunction. The factors associated with neurocognitive dysfunction were age, functional capacity, chronic heart failure, and hemoglobin levels. After the procedure, there was an overall improvement in the MoCA score of the patients, but 28% of the patients showed a reliable worsening of their condition. The factors associated with MoCA worsening were platelet transfusions and the amount of red blood cell units transfused.

**Conclusion:**

The correction of severe AS leads to an improvement in neurocognitive function after 2–3 months. This improvement does not differentiate between SAVR and TAVI after matching for pre-procedural factors. The only modifiable factor associated with pre-procedural neurocognitive function is anemia, and anemia correction with red blood cell transfusions is associated with a worsening of neurocognitive function. This leads to the hypothesis that anemia correction before the procedure (with iron and/or erythropoietin) may limit the risk of a post-procedural worsening of neurocognitive function.

## Introduction

Aortic valve stenosis (AS) is the most common heart valve disease in the elderly. Patients >75 years have a prevalence rate of AS of 12.4% and that of severe AS of 3.4% (1). Presently, the main therapeutic options for severe AS are surgical aortic valve replacement (SAVR) and transcatheter aortic valve implantation (TAVI). Many studies have compared the clinical outcomes of both techniques in terms of mortality and complications after the procedures. Conversely, a neurocognitive assessment after the procedures on those with severe AS has been done only in a few studies, often with a limited patient population (2–6).

So far, the results produced have either remained limited or conflicting in nature, and there is a gap in knowledge with respect to the factors that may influence the changes in neurocognitive function after the procedures. One certain factor is that elderly (>70 years) patients referred to AS procedures have a pre-procedural impairment of neurocognitive function. When tested with a Montreal Cognitive Assessment (MoCA), 70% of the patients have a MoCA score <26, which is considered the cutoff value for normal cognitive function (7). This pattern finds different causative factors such as comorbidities, previous neurological events, dementia, and other neurological diseases; however, inadequate cerebral blood flow may be a contributor, especially in elderly subjects (8). As a matter of fact, both TAVI and SAVR imply a number of mechanisms that can affect neurocognitive function. Restoring adequate cerebral blood flow by removing valvular obstruction may, *per se,* determine an improvement in neurocognitive function. However, other factors may deteriorate neurocognitive function. In SAVR, the use of cardiopulmonary bypass induces an inflammatory reaction, and the open heart chamber procedure inevitably leads to air micro-emboli and possibly solid particle embolism (9). TAVI is associated with calcium embolism at the time of the aortic valve deployment and with patterns of solid particle embolization that correlate to aortic annulus calcification (10).

This multifactorial impact of procedures correcting severe AS is probably the major determinant of the changes observed in neurocognitive function.

The OUTcomes evaluation of current therapeutic STrategies for severe Aortic valve steNosis and the ageING population in ITALY (OUTSTANDING ITALY) is a registry study performed by the Cardiologic Network of the Italian Clinical Research Hospitals (IRCCS). Within this registry, there are different branches exploring a number of outcome variables in procedures correcting severe AS. The neurocognitive branch explores both pre- and post-procedural changes in neurocognitive function in patients aged 65–80 years. The present study addresses the pre-procedural pattern of neurocognitive function, the effects of the procedure on neurocognitive changes, and the factors associated with these changes.

## Methods

All patients were prospectively enrolled in the OUTSTANDING ITALY registry, which is funded by the Italian Ministry of Health. The study was approved by the local ethics committee of San Raffaele Hospital (OSR 14/12/2017 protocol number 298/2017) and subsequently amended to include a neuropsychological function assessment. All patients provided written informed consent for participation.

### Patient population and data collection

All patients were enrolled at the IRCCS Policlinico San Donato. The minimum age for participation in the study was fixed at 66 years. The data collection process included the pre-procedural, procedural, and post-procedural factors incorporated in the OUTSTANDING ITALY registry. Neuropsychological function was assessed the day before the procedure and at the follow-up visit 2–3 months after the procedure. Professional psychologists (LR and LB) provided the cognitive assessment, which was based on the MoCA test.

The MoCA is a screening test designed to evaluate cognitive function and the presence of mild cognitive impairment (MCI) (11, 12). It consists of 30 items that assess multiple cognitive domains: memory (immediate and delayed recall), visuospatial abilities, executive functions, attention, concentration, language, and spatial and temporal orientation. The MoCA test yields a total score of 30, with a cutoff value of 26, which is indicative of normal functioning. However, the literature suggests a second cutoff of 23, reporting greater specificity (95%) and sensitivity (96%) for detecting cognitive impairment (13).

The MoCA-MIS (Memory Index Score) represents a subscore of the delayed recall memory test, which is able to discriminate between coding and recalling difficulties. It is also a useful predictor of the possible evolution of mild and neurocognitive disorders (14). The MIS score ranges from 0 to 15 points, with the cutoff set at 7 points: 3 points are assigned for each spontaneously recalled word (5 in total), 2 points if the recall occurs by semantic cues, 1, if it occurs by multiple choice cues, and 0 points, are assigned in the absence of a recall.

A total of 206 patients were enrolled in the pre-procedural phase, 99 of whom completed the post-procedural neurocognitive screening 2–3 months later.

### Statistics

Data are expressed as the mean (standard deviation), the median (interquartile range), or the number (%). Differences between binary variables were assessed by performing a univariate analysis using Pearson's chi-square test. Differences between continuous variables were assessed using paired (within-group differences) and unpaired (between-group differences) Student's *t*-tests. Regression analysis was used to correlate continuous variables. A sensitivity analysis comparing SAVR and TAVI patients was performed through propensity matching.

The primary outcome measure of the study was the difference between pre-procedural and follow-up MoCA parameters. To identify patients with a clinically relevant decrease in neurocognitive function, the pre-/post- procedural changes in the MoCA score were expressed as a categorical variable (improved, unchanged, and worsened). This categorization was performed using the Reliable Change Index (RCI). Briefly, the RCI is calculated as [(X2 − X1) − (M2 − M1)]/SED, where X1 = observed first test score, X2 = observed second test score, M1 = group mean first test score, M2 = group mean second test score, and SED = standard error of the difference corrected for the test–retest correlation coefficient *r* (5, 15). The RCI describes a confidence interval around the mean differences in scores. For all the RCIs, a reliable change was calculated by setting the alpha value to 0.05 (two-tailed). Therefore, a “reliable improvement” was adjudicated for an RCI exceeding + 1.96 and a “reliable worsening” for an RCI below −1.96.

All statistical analyses were performed by using computerized packages (SPSS 20.0, IBM, Chicago, IL, USA; GraphPad, GraphPad Software, Inc, San Diego, CA, USA; and MedCalc, MedCalc Software, Ostend, Belgium). A two-tailed *P*-value<0.05 was considered significant for all the statistical tests.

## Results

**Figure 1:**
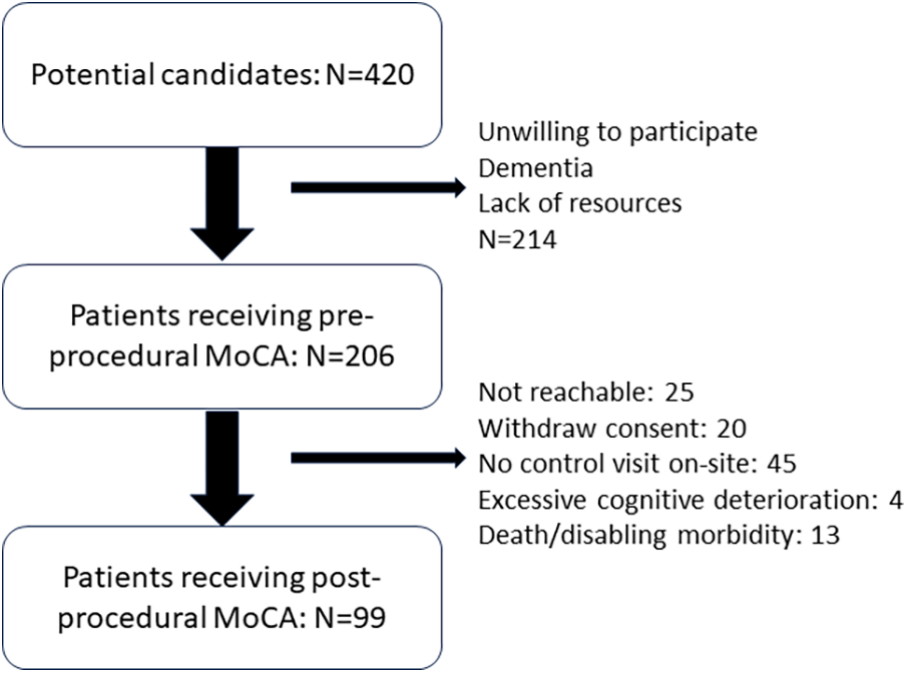
Patient flow and reasons for missing follow-up in the patient population.

The study period was between September 2022 and December 2023. During this period, 420 patients received either a TAVI or a SAVR for severe aortic stenosis. A total of 206 patients completed the pre-procedure evaluation. The median follow-up time was 66 days (interquartile range 51–89), and 99 patients completed the evaluation at follow-up. Figure 1 shows the determinants of this 50% dropout. The general characteristics of the patient population are reported in Table 1. The patients’ age ranged from 66 to 95 years. A total of 42 (20.4%) patients received a TAVI procedure and 164 (79.6%) a SAVR procedure. A number of comorbidities were present, the most frequent being diabetes, stable angina, syncope, and chronic heart failure. Data on pre-procedural neurocognitive function are reported in Table 2. Overall, the great majority of the patients (91%) had MoCA scores below the limit of normality of 26 points and 70% below the cutoff of 23 points. Univariate analysis demonstrated that a limited number of pre-procedural factors were associated with the MoCA score; these were age (correlation coefficient −0.312, *P* = 0.001), hemoglobin (correlation coefficient 0.152, *P* = 0.026), New York Heart Association (NYHA) class (correlation coefficient −0.184, *P* = 0.029), and chronic heart failure. When pooled together in a linear multivariable regression analysis, only age remained independently associated with the MoCA score (regression coefficient −0.182, *P* = 0.001).

**Table 1:** General characteristics of the patient population (*n* = 206).

Factor	Value
Age (years)	80.2 (6.5)
Sex (male patients)	96 (46.6)
Weight (kg)	72.5 (14.1)
Body mass index (kg/m^2^)	26.8 (5.1)
Systolic arterial pressure (mmHg)	131 (22)
Hemoglobin (g/dl)	13.1 (1.6)
Serum creatinine (mg/dl)	1.15 (0.86)
Chronic obstructive pulmonary disease	25 (12.1)
Diabetes	57 (27.7)
Chronic heart failure	27 (13.1)
Left ventricular ejection fraction (%)	58.8 (11.6)
Stroke or transient ischemic attack	23 (11.2)
Syncope	25 (12.1)
Previous myocardial infarction	24 (11.7)
Stable angina	29 (14.1)
Unstable angina	3 (1.5)
Previous vascular surgery	9 (4.0)
Previous cardiac surgery	15 (7.3)
NYHA class	2 (1–2)
Smoking history	98 (47.6)
Education (years)	8 (5–13)
Surgical aortic valve replacement	42 (20.4)
Transcatheter aortic valve implantation	164 (79.6)

Data are presented as mean (standard deviation), median (interquartile range), or number (%).

**Table 2:** Pre-procedural neurocognitive assessment (*N* = 206).

Parameter	Value (points)
MoCA total score (out of 30)	20.3 (3.9)
MoCA components	
Visuospatial/executive	2.6 (1.2)
Naming	2.7 (0.5)
Memory	1.6 (1.5)
Attention	4.7 (1.2)
Language	1.7 (0.7)
Abstraction	0.96 (0.77)
Orientation	5.5 (0.8)
MoCA score <26/30	187 (90.8)
MoCA score <23/30	144 (69.9)
Memory index score	8.7 (3.4)

Data are presented as mean (standard deviation) or number (%).

**Figure 2:**
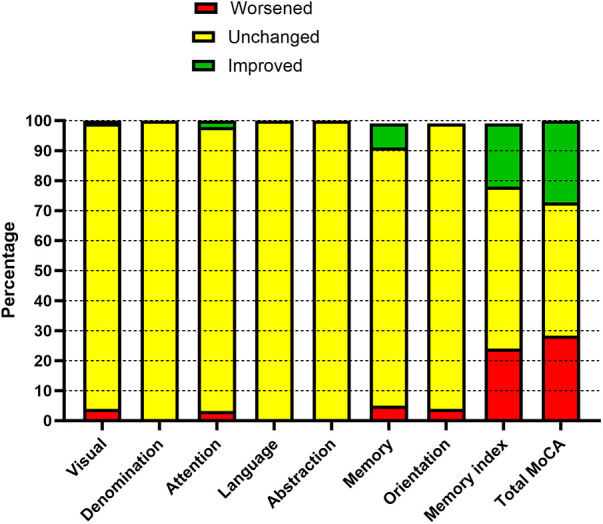
Reliable changes in cognitive function before and after the procedure (2–3-month follow-up).

Table 3 reports the neurocognitive changes that occur 2–3 months after the procedure. In total, 99 patients completed the follow-up; of them, 22 received a SAVR, and 77 received a TAVI. The total MoCA score improved significantly (*P* = 0.001); all its components improved with the exception of orientation, but the improvement was statistically significant only for visuospatial and memory components. The MIS improved significantly. The distribution of patients according to the RCI is reported in Figure 2. Overall, the largest changes were observed with respect to the memory components; 27 patients (27.3%) showed an improvement in terms of the RCI, 28 (28.3%) experienced worsening, and 44 (44.4%) experienced no changes.

**Table 3:** Pre-/post-neurocognitive changes in patients with follow-up (*N* = 99).

Parameter	Pre-procedure	Post-procedure (2 months)	Mean difference (95% CI)	*P*
MoCA total score (/30)	20.4 (4.1)	21.7 (4.2)	1.32 (0.86 to 1.79)	0.001
MoCA components				
Visuospatial/executive	2.6 (1.3)	3.0 (1.24)	0.39 (0.20 to 0.59)	0.001
Naming	2.7 (0.5)	2.8 (0.5)	0.10 (−0.007 to 0.21)	0.068
Memory	1.6 (1.5)	2.1 (1.6)	0.46 (0.17 to 0.74)	0.002
Attention	4.6 (1.3)	4.8 (1.15)	0.15 (−0.55 to 0.35)	0.150
Language	1.6 (0.7)	1.7 (0.79)	0.07 (−0.06 to 0.21)	0.288
Abstraction	0.96 (0.77)	1.1 (0.78)	0.14 (−0.02 to 0.30)	0.085
Orientation	5.5 (0.8)	5.5 (1.00)	−0.03 (−0.20 to 0.13)	0.716
MoCA score <26/30	87 (87.9)	82 (82.8)	Not applicable	0.001
MoCA score <23/30	69 (69.7)	49 (50.5)	Not applicable	0.001
Memory index score	8.5 (3.5)	9.9 (3.6)	1.35 (0.83 to 1.9)	0.001

CI, confidence interval.

Data are presented as mean (standard deviation) or number (%).

Worsening of the RCI was defined as neurocognitive deterioration and tested for an association with pre-procedure factors and with the outcome data (Table 4). The only factors that were associated with neurocognitive failure were the total amount of red blood cells transfused (units) and the need for platelet transfusions (yes/no). Patients receiving platelet transfusions experienced significantly (*P* = 0.038) higher periprocedural bleeding (650 ± 707 ml/12 h) compared with those who did not (290 ± 207 ml/12 h). No pre-procedural factor was associated with neurocognitive failure. Patients who experienced a pre-procedural stroke (11 patients) had a neurocognitive failure in 4 (36.4%) cases, those who did not (88 patients) had a neurocognitive failure in 24 (27.3%) cases (*P* = 0.528).

**Table 4:** Outcome data in patients with and without neurocognitive deterioration (*N* = 99).

Outcome variable	Cognitive function preserved or improved (*N* = 71)	Cognitive function deteriorated (*N* = 28)	*P*
Perioperative myocardial infarction	1 (1.4)	1 (3.4)	0.502
Surgical revision	0 (0)	1 (3.4)	0.289
Low cardiac output	4 (5.7)	0 (0)	0.318
Atrial fibrillation (new onset)	16 (22.9)	6 (20.7)	0.813
Pacemaker implantation	5 (7.1)	2 (6.9)	0.965
Left bundle branch block (new onset)	4 (5.7)	1 (3.4)	0.639
Ventricular arrhythmias	10 (14.3)	3 (10.3)	0.750
Acute kidney injury	9 (12.9)	7 (24.1)	0.165
Perioperative stroke	0 (0)	0 (0)	1.000
Post-procedure delirium	0 (0)	0 (0)	1.000
Pulmonary embolism	0 (0)	0 (0)	1.000
Gastrointestinal bleeding	1 (1.4)	0 (0)	0.525
Systemic infection	0 (0)	0 (0)	1.000
Peak serum creatinine (mg/dl)	1.06 (0.8)	1.37 (1.6)	0.187
Post-procedural bleeding (ml/24 h)	307 (219)	304 (311)	0.972
Red blood cell transfusions	11 (15.7)	3 (10.3)	0.752
Units of red blood cells transfused	1.5 (0.85)	3.3 (2.1)	0.037
Need for platelet transfusions	0 (0)	3 (10.3)	0.023

Data are presented as mean (standard deviation) or number (%).

In a subanalysis, the 22 patients undergoing SAVR were cross-matched with 22 TAVI patients using a propensity score based on age and pre-procedural MoCA. The pre-/post-procedural changes in the MoCA in this subgroup are reported in Table 5. The two groups were comparable for the pre-procedural factors associated with the MoCA score (age, NYHA class, hemoglobin, and chronic heart failure) and for the different components of the MoCA. At follow-up, the total MoCA score and its components did not significantly differ between the two groups.

**Table 5:** Pre-/post-clinical and neurocognitive changes in patients with follow-up (*N* = 99).

Parameter	Pre-procedure		*P*	Follow-up (2 months)		Mean difference (95% CI) at follow-up	*P*
	TAVI	SAVR		TAVI	SAVR		
Age (years)	75.1 (4.8)	72.5 (4.6)	0.077	N/A		N/A	N/A
Hemoglobin (mg/dl)	12.9 (1.5)	13.7 (1.6)	0.113	N/A		N/A	N/A
NYHA class	2 (1–2)	2 (1–2)	0.767	N/A		N/A	N/A
Chronic heart failure	16 (73)	21 (96)	0.100	N/A		N/A	N/A
MoCA total score (out of 30)	21.8 (3.1)	22.2 (4.3)	0.718	23.2 (3.4)	23.8 (3.8)	0.59 (−1.6 to −2.7)	0.591
MoCA components							
Visuospatial	2.9 (1.0)	3.2 (1.3)	0.356	3.1 (1.1)	3.6 (1.3)	0.32 (−0.41 to 1.04)	0.382
Naming	2.7 (0.5)	2.7 (0.5)	1.000	2.9 (0.3)	2.8 (0.4)	−0.09 (−0.30 to 0.12)	0.391
Memory	2.2 (1.5)	1.9 (1.7)	0.578	2.4 (1.6)	2.7 (1.6)	0.32 (−0.64 to 1.28)	0.510
Attention	4.8 (1.1)	4.5 (1.3)	0.510	5.2 (1.0)	4.8 (1.1)	−0.39 (−1.10 to 0.26)	0.237
Language	1.6 (0.7)	2.0 (0.7)	0.080	1.9 (0.8)	1.8 (0.7)	−0.02 (−0.51 to 0.48)	0.929
Abstraction	1.1 (0.7)	1.0 (0.8)	0.689	1.1 (0.9)	1.4 (0.7)	0.23 (−0.11 to 0.84)	0.128
Orientation	5.6 (0.6)	5.9 (0.5)	0.160	5.8 (0.5)	5.8 (0.4)	0.05 (−0.24 to 0.33)	0.748
Memory index score	9.4 (3.6)	9.6 (3.4)	0.866	10.5 (3.2)	11.3 (3.3)	0.77 (−1.22 to 2.77)	0.773

N/A, not applicable.

Data are presented as mean (standard deviation), median (interquartile range), or number (%). *N* = 22 for both propensity-matched groups.

## Discussion

In this study, a large cohort of patients with severe AS, referred to as either TAVI or SAVR, was tested for cognitive function. A total of 206 patients took a MoCA test immediately after the procedure, and 99 (48%) underwent the same evaluation after a median follow-up of 2 months. The rate of patients lost to follow-up is common in this kind of studies. Knipp et al. (16), in a series of 64 patients receiving transapical TAVI with a mean age of 73 years, reported an 80% rate of patients available for a 3-month follow-up. In an older (80-year-old) patient population undergoing TAVI, Auffret et al. (5) reported a follow-up failure rate of 50%, which was similar to that in our series for a similar mean age.

The 206 patients who underwent a pre-procedural test had a mean MoCA score of 20.3 and a rate of cognitive impairment (cutoff of 23/30) of 70%. This pattern was more severe than that reported by other authors (MoCA at baseline 22–25 points), but similar to that reported by a few others (3–5), all of whom included only TAVI patients. We are not aware of any study that assesses the determinants of the pre-procedural MoCA score, even if preoperative cognitive performance predicts clinical stroke and mortality after SAVR (17). In our series, the factors negatively associated with the MoCA score were age, hemoglobin, functional capacity, and chronic heart failure. Within these factors, the only modifiable component is the hemoglobin value. In the setting of chronic heart failure and cardiac surgery, treatment with iron supplementation and/or erythropoietin has been suggested as an effective measure to improve the outcome (18). There are no studies in the specific setting of a SAVR or TAVI procedure for severe AS; our findings generate the hypothesis that pre-procedural anemia correction may be beneficial in improving the cognitive status before and after the procedure. Low values of hemoglobin induce a decrease in the arterial oxygen content and oxygen delivery to the brain, which may justify poor levels of neurocognitive function.

Overall, the MoCA test result showed a significant improvement in the condition of patients at follow-up. This was in agreement with that of other studies using the MoCA or other cognitive function tests in the TAVI procedure (4, 5, 16, 19), but not with that of other studies where the cognitive function remained unchanged at 1–6 months of follow-up (3, 20). A meta-analysis demonstrated that cognitive function significantly improved 1 month after TAVI but not after 3 or 6 months (21). Considering our median time of follow-up, our data seem to confirm this finding. Of course, it must be noted that patients with TAVI are generally older than 80 years and that a worsening of neurocognitive function after 6 months may be justified simply by the well-known effects of aging. Limited data exist for cognitive function changes occurring after SAVR. De Rui et al. (7) showed a significant worsening of the patients’ condition in the MoCA test performed 1 year after SAVR; however, this finding is not confirmed in our series. Basically, our data support the concept that the removal of aortic valve obstruction is beneficial in neurocognitive terms, regardless of the procedure.

Even if the mean neurocognitive function improves, there are a non-trivial number of patients who show a reliable worsening of their condition in the MoCA test (28%). Of note, this pattern is not associated with pre-procedural factors, whereas some outcome factors (for example, the total amount of RBC transfused and the need for platelet transfusion) are significantly more prevalent in patients who suffer a worsening of cognitive function. This may be a marker for a more complex post-procedural outcome and/or procedural bleeding, especially in SAVR. However, a direct effect of RBC transfusions on cognitive function cannot be excluded, as suggested by other studies in cardiac surgery (22, 23) and other surgeries (24). In addition, RBC transfusions may be a marker of low levels of hemoglobin, which, in turn, are determinants of pre-procedural low MoCA scores. This again leads to the hypothesis that a pre-procedural optimization of hemoglobin values may be an important factor in reducing the risk of post-procedural cognitive decline. The role of platelet transfusions could be interpreted as a direct negative effect or as a surrogate for a bleeding episode, with both being expressions of a complicated post-procedural course. In this study, the patients who received platelet transfusions experienced a significantly high amount of bleeding.

An important finding of our study is that the memory domain plays a major role both as a determinant of improved neurocognitive function and as the main factor determining worsening.

Memory is a complex and multifaceted cognitive domain that is most commonly affected in cases of MCI (25). Gathering or assimilating new information not only requires the ability to store but also the use of strategies aimed at facilitating the memorization process. The underlying mechanisms are influenced by other cognitive abilities, such as executive functions and attentional abilities, and are therefore indicative of global functioning. Shin et al. (26) showed how an improvement in memory and executive functions in patients with MCI leads to an increase in global cognitive function; our data seem to confirm these results. Our research suggests an increase in autonomous recall abilities after aortic procedures (SAVR or TAVI), as well as in cue recall. The MoCA-MIS score enhancement indicates a better ability to encode information in the post-procedural phase compared with the pre-procedural phase, with a lower propensity to forgetfulness and slipping into an amnesiac state (27). Memory improvements may be the result of the restoration of cardiac output and improved hemodynamic status following TAVI or surgical procedures (28). This can lead to an increase in cerebral blood flow and oxygenation in the brain areas responsible for these processes (29).

Furthermore, cerebral hypoperfusion and low cardiac output have been shown to be primarily linked to cognitive decline, especially in the domains of attention and executive function (28). Hypothetically, the restoration of cerebral blood flow could be responsible for the improvement of these cognitive functions, as revealed in our data (28, 30).

Finally, the literature shows that patients with severe AS typically present a high prevalence of vascular risk factors associated with the possible development of vascular cognitive impairment affecting the frontal lobes. Delayed recall memory, attention, and executive function are domains dependent on these brain structures. For this reason, they may see improvement because of hemodynamic changes, in addition to worsening cases of adverse events such as microembolic lesions (28).

This study is not a targeted one and is certainly underpowered to detect differences in neurocognitive outcomes in SAVR vs. TAVI. However, in a propensity-matched subanalysis, no differences were detected between the two procedures. In a series of 100 patients undergoing transfemoral TAVI vs. SAVR, no differences in early (3 days) post-procedural cognitive function were detected, with both groups showing a worsening (2). A prospective trial of neurocognitive outcomes in patients undergoing TAVI vs. SAVR is ongoing, but the results are not available yet (31).

In conclusion, our study demonstrates that the correction of severe AS determines a significant improvement in neurocognitive function at a 2–3-month follow-up; a worsening of neurocognitive function (for example in the memory domain) affects a non-trivial rate (28%) of patients. The main factors associated with low MoCA scores before and after the procedure are related to anemia and its correction with RBC transfusions. This highlights the role of pre-procedural anemia correction in reducing the risk of neurocognitive deterioration.

## Data Availability

The raw data supporting the conclusions of this article will be made available by the authors without undue reservation.
